# Bright Excitonic Fine Structure in Metal-Halide Perovskites: From Two-Dimensional to Bulk

**DOI:** 10.1021/jacs.3c11957

**Published:** 2024-02-07

**Authors:** Katarzyna Posmyk, Natalia Zawadzka, Łucja Kipczak, Mateusz Dyksik, Alessandro Surrente, Duncan K. Maude, Tomasz Kazimierczuk, Adam Babiński, Maciej R. Molas, Wakul Bumrungsan, Chanisara Chooseng, Watcharaphol Paritmongkol, William A. Tisdale, Michał Baranowski, Paulina Plochocka

**Affiliations:** † Department of Experimental Physics, Faculty of Fundamental Problems of Technology, Wroclaw University of Science and Technology, Wroclaw 50-370, Poland; ‡ Laboratoire National des Champs Magnétiques Intenses, EMFL, CNRS UPR 3228, Université Grenoble Alpes, Université Toulouse, Université Toulouse 3, INSA-T, 38042 Grenoble, Toulouse 31400, France; § Institute of Experimental Physics, Faculty of Physics, University of Warsaw, Warsaw 02-093, Poland; ∥ Department of Materials Science and Engineering, School of Molecular Science and Engineering, Vidyasirimedhi Institute of Science and Technology (VISTEC), Rayong 21210, Thailand; ⊥ Department of Chemical and Biomolecular Engineering, School of Energy Science and Engineering, Vidyasirimedhi Institute of Science and Technology (VISTEC), Rayong 21210, Thailand; # Department of Chemical Engineering, Massachusetts Institute of Technology, Cambridge, Massachusetts 02139, United States; ¶ Department of Chemistry, Massachusetts Institute of Technology, Cambridge, Massachusetts 02139, United States

## Abstract

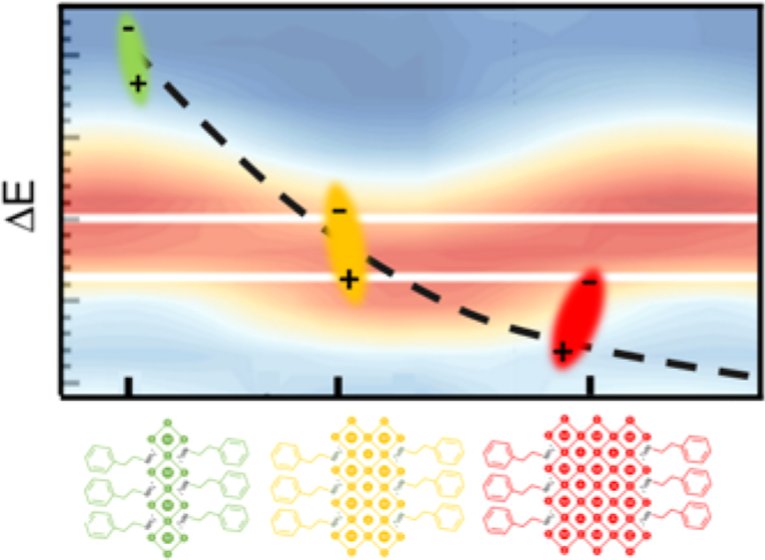

The optical response of two-dimensional (2D) perovskites, often referred to as natural quantum wells, is primarily governed by excitons, whose properties can be readily tuned by adjusting the perovskite layer thickness. We have investigated the exciton fine structure splitting in the archetypal 2D perovskite (PEA)_2_(MA)_*n*−1_Pb_*n*_I_3*n*+1_ with varying numbers of inorganic octahedral layers *n* = 1, 2, 3, and 4. We demonstrate that the in-plane excitonic states exhibit splitting and orthogonally oriented dipoles for all confinement regimes. The evolution of the exciton states in an external magnetic field provides further insights into the *g*-factors and diamagnetic coefficients. With increasing *n*, we observe a gradual evolution of the excitonic parameters characteristic of a 2D to three-dimensional transition. Our results provide valuable information concerning the evolution of the optoelectronic properties of 2D perovskites with the changing confinement strength.

## Introduction

Excitons represent fundamental (interband) electronic excitation in a semiconductor. The role of this Coulomb-bound electron–hole pair is particularly important for quantum confined systems, where electrostatic and exchange interactions are enhanced, strongly affecting the transitions’ energy spectrum and the selection rules.^1−7^ Therefore, a detailed understanding of the excitonic properties of such nanostructures is crucial to their use in optoelectronic and photonic devices. For decades, confined systems served as platforms to investigate fundamental exciton physics.^1,8−12^ Recently, two-dimensional (2D) van der Waals crystals^13−16^ have remarkably enriched this excitonic playground, essentially due to the combined spatial and dielectric confinement effects, which are particularly strong. A beautiful illustration of this is given by 2D metal-halide perovskites,^14,17,18^ where tightly bound excitons (with exciton binding energy reaching several hundred meV) open the path to investigate light–matter interaction in a strong coupling regime far above cryogenic temperatures^19,20^ or considerably pronounced exciton fine structure.^3,6,21,22^

In 2D perovskites, the degeneracy of excitonic states with respect to the angular momentum is often completely lifted,^3,5,6,24^ due to enhanced exchange interactions and the low lattice symmetry. The fine structure splitting (FSS) of excitonic states can reach values of a few up to tens of meV.^3,4,6,25^ This is orders of magnitude larger than in “classical” III–V semiconductor nanostructures,^1,11,26^ making 2D perovskites a very attractive medium to study^3−6,22,25,27−29^ and (potentially) use the exciton fine structure.^30−32^

**Figure 1:**
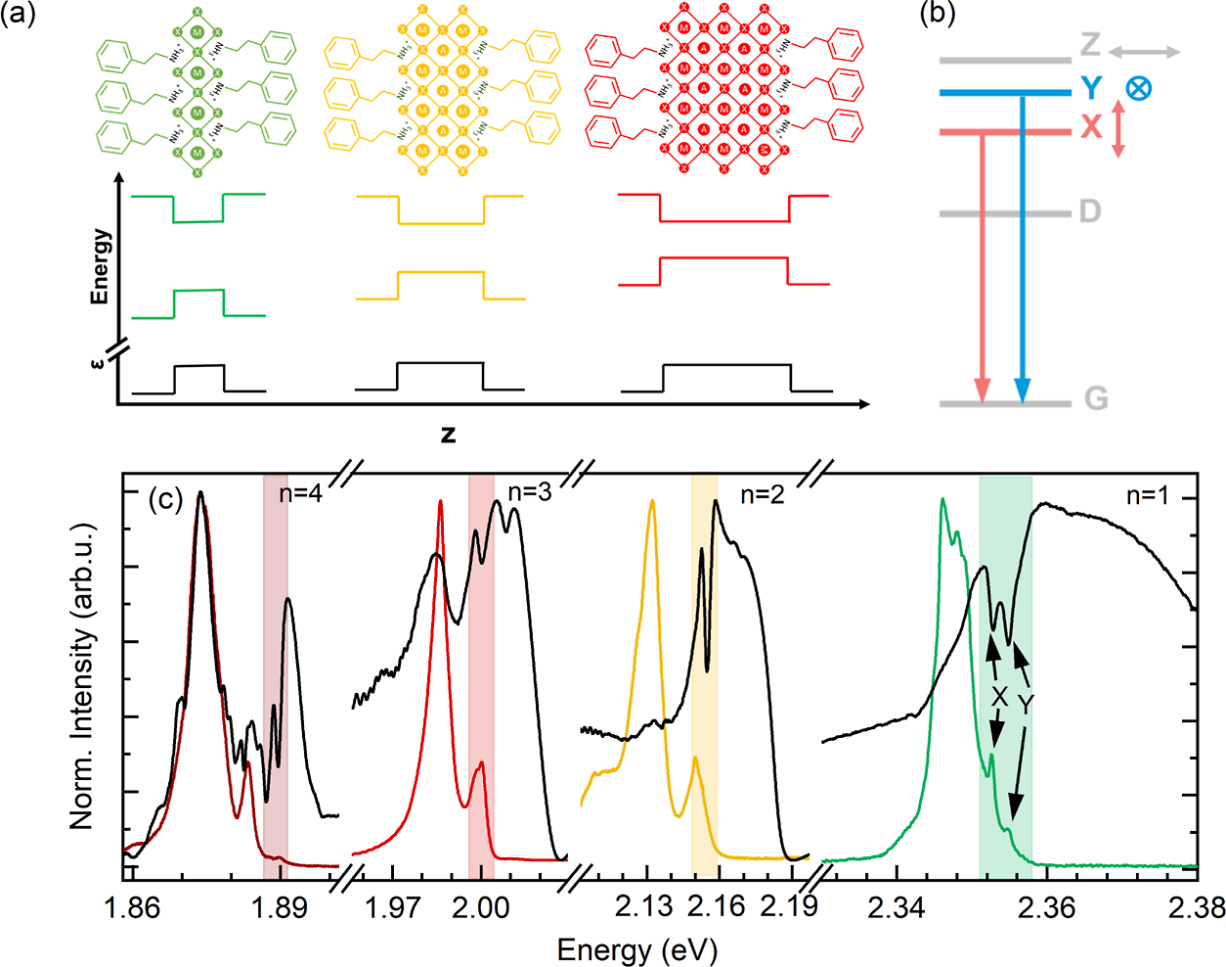
(a) Schematic view of a single layer of 2D perovskites with *n* = 1, 2, 3 inorganic layers, together with the band alignment and dielectric screening profile, which provide quantum and dielectric confinement. (b) Scheme of the band-edge exciton fine structure for PEA_2_(MA)_*n*−1_Pb_*n*_I_3*n*+1_ 2D perovskites. G denotes a ground state of the system (no exciton) and X and Y are the linearly polarized orthogonal states relevant to this work; Z and D complete the exciton manifold, where D = dark state, and Z = bright state polarized out of the plane of the crystal. (c) Photoluminescence (PL) (colored lines) and reflectance (black lines) spectra for samples with varying thicknesses of inorganic layers—*n*. Shaded areas indicate the excitonic transitions. For *n* = 1, the arrows indicate the two in-plane bright exciton states. The multiple resonance structures visible in the reflectance spectra for *n* = 3 and *n* = 4 are attributed to the phonon replica of the main excitonic transition. A more detailed discussion can be found in the Supporting Information.

The structure of 2D perovskites can be seen as natural quantum wells, where slabs of metal-halide octahedral units are surrounded from both sides by large organic cations, as schematically shown in Figure 1a. 2D perovskites form type-I quantum wells, where charge carriers are confined in the octahedral slab, and the organic spacers act as the barrier, which provides both spatial and dielectric confinement.^14,33,34^ Such organic–inorganic stacks, belonging to the Ruddlesden–Popper phase of the 2D perovskite subgroup,^14,17^ are described by the general formula L_2_A_*n*–1_M_*n*_X_3*n*+1_, where L is a large organic monovalent cation, A is a small monovalent cation, M is a metal atom, X is a halide atom, and *n* denotes the number of octahedral layers. The exciton binding energy in 2D perovskites can reach several hundreds of millielectronvolts for the thinnest quantum wells (*n* = 1) and can be decreased to as low as several meV when tuning between the 2D and three-dimensional (3D) limits by controlling the quantum well thickness.^18,21,35−37^

In metal-halide perovskites, we can distinguish four band-edge exciton states, which differ in their total angular momentum, and their out-of-plane projection, namely, a bright triplet and dark singlet state.^7,38−40^ The exchange interaction between the spins of the electrons and holes always splits the bright states and dark exciton states. Depending on the symmetry of the system, the degeneracy of the bright states can be either partially or completely lifted.^7,40,41^ In the latter case, the dipoles of the three split bright states are linearly and orthogonally polarized. The fine structure of the ground exciton state is schematically presented in Figure 1b. This excitonic fine structure has been experimentally established for the orthorhombic phase of bulk and perovskite nanocrystals^7,12,32,38,42,43^ as well as in the 2D perovskite (PEA)_2_PbI_4_.^3−6,22,24^

It is perhaps surprising that, despite the easy tunability, the impact of the quantum confinement on the exciton fine structure in 2D perovskites has not been investigated so far, even though it appears to be crucial to understand the high emission efficiency of these materials.^4,38,44^ This is even more intriguing, considering that the FSS of bright exciton states has already been observed in bulk metal-halide perovskites,^23^ revealing the evolution of the electronic properties between the two extrema of the quantum confinement regime (*n* = 1 and *n* = ∞). It is noteworthy that the quantitative understanding of the evolution of the excitonic structure is not straightforward. The increase in the inorganic layer thickness not only alters the quantum confinement but is also coupled with changes in dielectric conditions and carrier mass.^18,35^ Consequently, a robust experimental benchmark is crucial for developing any exciton theory bridging the gap between 2D and 3D limits.

Here, we address this challenge by focusing on the bright (in-plane) exciton fine structure in archetypal 2D perovskite (PEA)_2_(MA)_*n*−1_Pb_*n*_I_3*n*–1_ (where PEA stands for phenylethylammonium and MA stands for methylammonium), often referred to as PEPI. With the use of polarization-resolved and magneto-optical spectroscopy, we investigated the evolution of the bright in-plane exciton state splitting as a function of the inorganic octahedral layers’ thickness (*n* = 1, ..., 4). We find that the in-plane state splitting systematically decreases with increasing *n*, reaching the value expected for bulk perovskites already for *n* = 4. We further determine the evolution of the bright exciton *g*-factors, which also systematically approach the bulk values with increasing quantum well thickness. The extracted parameters can be used as a benchmark for the band structure and exciton state modeling because they contain information concerning the anisotropy and dispersion of the bands.

## Results

The investigated high-quality (PEA)_2_(MA)_*n*−1_Pb_*n*_I_3*n*+1_ (*n* = 1, ..., 4) crystals were grown by a cooling-induced crystallization method^45,46^ with an average crystal size of a few millimeters (Figure S1). The high phase purity of these crystals was confirmed by powder X-ray diffraction (Figure S2), which shows clean diffraction patterns of their respective phases. Optical spectra, namely, microphotoluminescence (μPL) and microreflectance (μR) were taken in the backscattering geometry at temperature *T* = 4.2 K. The measurements were performed using an objective having a numerical aperture (NA) of 0.55, selectively sensitive to the states with an in-plane orientation. Typical spectra of PL and reflectance (*R*) measured from the samples with *n* = 1, ..., 4 are shown in Figure 1c. With increasing *n*, the PL spectrum and exciton resonances (indicated by shaded areas) visible in the reflectance spectra shift toward lower energies—an indication of decreasing quantum and dielectric confinement.^18,35^ We notice that for all samples, the PL spectra show a complex line shape with two main features. The dominating PL features are red-shifted with respect to the resonant excitonic features of reflectance spectra, while the less intense PL peaks overlap with them (shaded areas in Figure 1b). Based on aligning of high-energy PL peaks with the excitonic transitions observed in reflectance spectra, we attribute them to the free exciton recombination^3^ (see also a more extended discussion presented in the Supporting Information). At the same time, the more intense, red-shifted PL peak can be attributed to local potential variation, related to shallow trap states,^47^ to band gap fluctuation,^48,49^ or to polaronic effects.^50,51^ Evidently, the relaxation processes are faster than the free exciton recombination, because in each sample, the PL is dominated by a low-energy part related to excitons trapped at extrinsic defects^47,51−53^ or local lattice reconfiguration.^48−50,54^ The detailed origin of the complex PL spectrum is beyond the scope of this work. From now on, we focus on the analysis of the reflectivity response, which unequivocally probes the energy associated with free excitonic transitions.

For the case of (PEA)_2_PbI_4_ (thinnest quantum well, *n* = 1), the significant splitting (∼2 meV) of the two in-plane (X and Y) states has been already reported^3,6,22,29^ and is easily observed both in the reflectance and PL spectra even without the use of polarization optics, as shown in Figure 1c (indicated by the arrows). For the remaining three samples (*n* = 2, 3, and 4), to reveal the FSS of the bright exciton, we performed polarization-resolved reflectance measurements.

**Figure 2:**
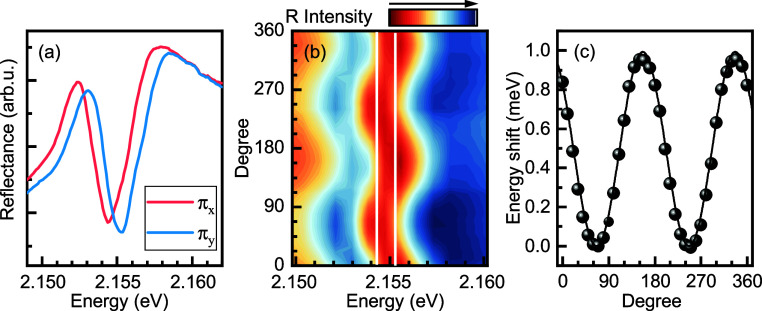
(a) Reflectance spectra measured from the sample with *n* = 2 in two orthogonal linear polarizations showing clear splitting between the two in-plane bright states of the exciton fine structure. Results for samples with *n* = 3 and *n* = 4 are shown in the Supporting Information. (b) Dependence of the reflectance spectrum for PEPI *n* = 2 versus polarization angle. White lines indicate the energies of the two states. (c) The energy difference between the two features in reflectance extracted with a differential method is described in detail in reference 23^23^ and Supporting Information. The black line is a cos^2^(*x*) fit to the data points.

Two reflectance spectra taken with two orthogonal linear polarizations π_X_ and π_Y_ for sample *n* = 2 are presented in Figure 2a. Similar spectra for the thicker 2D perovskites are shown in the Supporting Information (Figures S3–S5). The energy shift of the transition between the π_X_ and π_Y_ polarizations (red and blue curve) is the signature of bright exciton FSS,^3,6,23^ hidden in nonpolarization-resolved spectra, due to the broadening of the transitions. We then performed a full angular dependence of the reflectance spectra versus polarization angle shown in Figure 2b and observed the characteristic oscillating pattern with a 180° period, resulting from the varying contribution of the two orthogonally polarized excitonic states with slightly different energy. To precisely determine the value of the FSS, we analyze the shift of the resonance versus the analyzer angle. To accurately extract the small-energy shift, we use the method described in detail in the SI and in the literature.^23,55^ The energy shift dependence Δ*E* is shown in Figure 2c. It is well-fitted with the function Δ*E* = δ cos^2^(α + ϕ), where α is the detection angle, ϕ is the phase, and δ = 1.0 meV (for *n* = 2 sample) is the bright in-plane exciton FSS.

**Figure 3:**
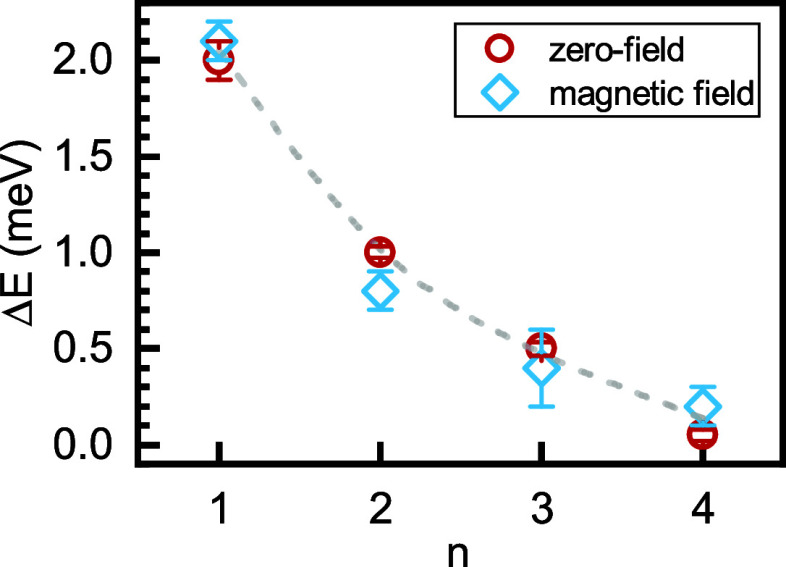
Energy splitting as a function of the number of inorganic octahedron layers *n*, extracted from polarization-resolved measurements without the magnetic field (circles) and from magneto-optical measurements (diamonds).

We applied the same procedure for the remaining crystals. The value of bright exciton FSS splitting δ versus the thickness of the quantum well is summarized in Figure 3 (red circles) and Table 1. The energy spacing between the in-plane excitonic states δ systematically decreases from ∼2 to ∼0.2–0.1 meV with increasing *n*. This can be qualitatively understood as the result of the decreasing exciton binding energy and the related increase of the extension of the exciton wave function in wider quantum wells.^35^ The increased distance between the electron and hole reduces the exchange interaction, which results in a decreasing bright exciton FSS.^2,26,42,56^ On a quantitative level, the provided values can serve as a benchmark for exciton models in metal-halide perovskites. For instance, for *n* = 4, the extracted δ ≈ 0.1 meV is comparable to the expected value for the bulk perovskite crystal (100–200 μeV^2,23,43^).

**Table 1:** Summary of the Parameters Extracted from the Measurements in the Magnetic Field^a^

	δ (meV)			
*n*	*B* = 0	*B* ≠ 0	g_∥_	*c*_0_ (μeV/T^2^)
1	2 (0.1)	2.1 (0.1)^3^	1.2^21^	0.36^21^
2	1 (0.03)	0.8 (0.1)	2.1	1.25
3	0.5 (0.04)	0.4 (0.2)	2.23	1.7
4	0.05 (0.03)	0.2 (0.1)	2.4	2.2

a Values of the energy splitting taken from the polarization-resolved measurements at zero field and extracted from the fitting with eq 3. Values in brackets are uncertainty estimations from fits.

**Figure 4:**
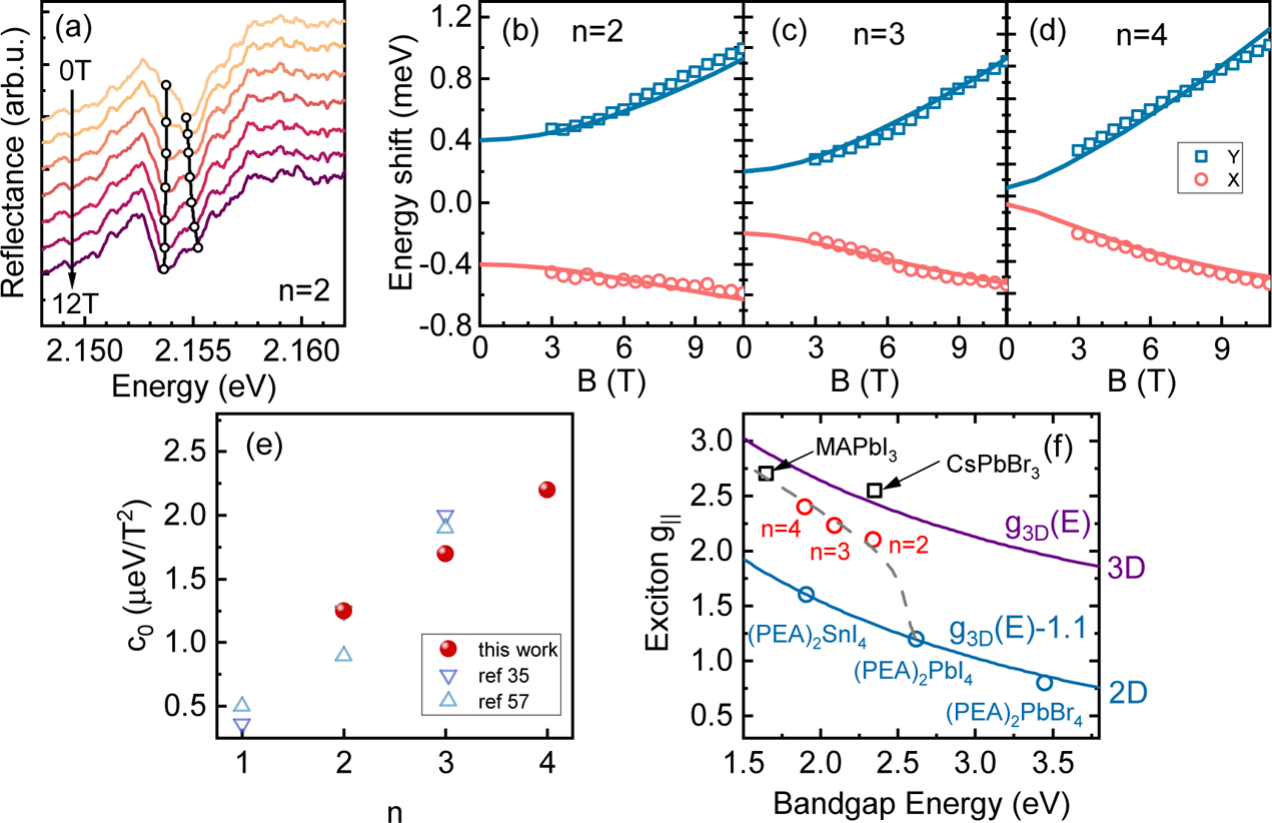
(a) Reflectance spectra for the *n* = 2 sample at different magnetic fields. Black lines track the shift of the excitonic transition as a function of the magnetic field. Results for samples with *n* = 3 and *n* = 4 are shown in the Supporting Information. Energy of the bright excitonic in-plane states with respect to the magnetic field for (b) *n* = 2, (c) *n* = 3, and (d) *n* = 4 samples. (e) Diamagnetic coefficient as a function of the number of inorganic octahedron layers *n*. Values obtained in references (35 and 57) are displayed for comparison. (f) Exciton *g*-factor in the direction parallel to the *c* axis of the crystal as a function of the band gap energy. Values of the bulk perovskite *g*_3D_(*E*) are taken after reference 58,^58^ and values of *g*-factors for 2D perovskites are taken after reference 4.^4^ The gray dashed line is a guide to the eye showing the evolution of *g*-factors from the 2D limit to the bulk limit.

To gain a deeper insight into the observed excitonic states, we measured the magnetoreflectance spectrum in the Faraday configuration (*B*∥*k*∥*c*) in static magnetic fields up to 12 T at temperatures *T* ≃ 10 K. As shown in Figure 4a, the magnetic field increases the splitting between the in-plane excitonic states. The observed evolution of the transition energy as a function of the magnetic field provides an additional measure of δ as well as exciton *g*-factor and diamagnetic shift.^1,3,23,36,41^ In the Faraday configuration, in-plane bright excitonic states are mixed with each other, gradually changing from linearly polarized at zero field to circularly polarized at high magnetic field.^1,23^ This effect is nicely visible in Figure 4a, where we show our measurements in the magnetic field on a circular polarization basis. At zero field, bright states are linearly polarized, therefore both are visible in the spectrum. However, with increasing field, as they gain a finite degree of circular polarization, one of the states becomes more prominent in one polarization (see also Figure S6 in the Supporting Information).

The energy *E*_Y/X_ of each exciton state X and Y in the presence of the exchange interaction and the magnetic field is described by
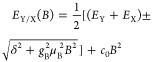
1where *B* is the magnetic field, μ_B_ is the Bohr magneton, and *g*_B_ is the bright exciton *g*-factor along the *c*-axis of the crystal given by the sum of the electron and hole *g*-factors
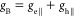
2*c*_0_ is the diamagnetic coefficient, which depends on the size of the exciton wave function.^35^ The opposite shifts (±) of X and Y transitions arise from the Zeeman effect.^1,23,41^ The transition-energy shift relative to the average energy of in-plane excitonic transition  at zero field is
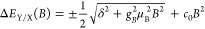
3

The evolution of the shift of the excitonic states in the magnetic field for samples *n* = 2, 3, and 4 (for a detailed analysis see Supporting Information) is summarized in Figure 4b–d (the dependence for *n* = 1 has already been published^3^). In the analysis, we omit the spectra below 3 T because excitonic states for samples *n* = 3 and 4 cannot be resolved very accurately at low fields. By fitting the data with eq 3, we extract the values of δ, *c*_0_, and *g*_B_ as a function of *n*. The dependence of δ(*n*) obtained with this approach is shown in Figure 3. We emphasize that the values extracted with the two different approaches (energy dependence on the magnetic field and linear polarization-resolved measurements at 0 field) are in very good agreement, further confirming the validity of our analysis.

The non-negligible influence of the diamagnetic shift is also visible in the presented data. The blue shift and the red shift of the upper and lower energy transitions are not symmetric, which reflects the quadratic term in eq 3. The extracted diamagnetic shift coefficients *c*_0_ increase with *n*, due to the increasing in-plane extension of the exciton wave function in thicker quantum wells^35^ (*c*_0_ ∼ ⟨*r*^2^⟩ where ⟨*r*^2^⟩ is a mean-square wave function extension^59^). The values of *c*_0_ are shown together with previously reported values^35,57^ in Figure 4e, and they show very good agreement with each other.

Our measurements also allow us to obtain the dependence of the bright exciton *g*-factors as a function of *n*. This is shown in Figure 4 (f) (red circles), together with the *g*-factors for other representatives of 3D (black squares) and 2D perovskites (blue circles).^4,21,60−62^ The values of the exciton *g*-factor for *n* = 2, 3, and 4 are exactly between the 2D (*n* = 1) and 3D cases and gradually approach the 3D value (MAPbI_3_), which points to a crucial influence of the confinement on the *g*-factor. The observed variation in the exciton *g*-factor aligns with the broad predictions of the **k**·**p** model for metal-halide perovskites.^58,60^ Consistent with expectations, the values of the *g*-factor decrease with the opening of the band gap. It is worth noting that the evolution of the *g*-factor as a function of the band gap shows the same curvature for 3D and 2D perovskites (*n* = 1). To illustrate this, we plot the **k**·**p** model prediction for the exciton *g*-factor in 3D perovskites after reference 58^58^ as a purple line. The same line, shifted downward by 1.1, perfectly describes the *g*-factors in PEA-based 2D perovskites (blue line). The change of the *g*-factor can be attributed to the enhanced mixing of electron bands with the split-off electron bands under a strong confinement regime as recently proposed for perovskite nanocrystals.^60^

## Conclusions

We studied the bright in-plane exciton FSS together with its *g*-factors for the PEA_2_(MA)_*n*−1_Pb_*n*_I_3*n*+1_ compounds with *n* varying from 1 to 4. We have shown that similar to pure 2D and 3D cases, the in-plane excitonic states exhibit splitting and orthogonally oriented dipoles under the intermediate confinement regime. For the first time, we have described the evolution of the exciton *g*-factors as a function of *n* for PEA_2_(MA)_*n*−1_Pb_*n*_I_3*n*+1_. The observed FSS, together with the exciton *g*-factors, approaches the value characteristic of bulk metal-halide perovskite as *n* increases. The evolution of both quantities can be understood as a result of the decreasing confinement. The parameters provided form a solid basis for further studies of the band structure and excitons in lead-halide perovskites, in particular, the role of quantum and dielectric confinement.

## Experimental Methods

### Synthesis and Sample Preparation

The synthesis of the samples was done using the cooling-induced crystallization method, described in detail in references 45 and 46.^45,46^ The high phase purity of these samples was confirmed by a powder X-ray diffraction technique using a Bruker D8 ADVANCE diffractometer.

### Optical Measurements

For PL and reflectance measurements without the use of a magnetic field, the samples were mounted on the cold finger of a He flow optical cryostat. All of these measurements were performed at 4.2 K.

For the microreflectance measurements, white light was provided by a tungsten halogen light source by Ocean Optics. The excitation and signal collection were done by using a long working distance microscope objective with 50× magnification and an NA of 0.55. The optical signal was analyzed with a 500 mm long monochromator with a grating of 1800 grooves per mm and detected with a liquid-nitrogen-cooled CCD camera. We mounted a half-wave plate in the detection path of our setup, in front of a linear polarizer oriented along the preferred polarization of the spectrometer grating. Magnetoreflectance measurements were performed at *T* = 10 K in a superconducting magnet in static magnetic fields up to 12 T. We used a 515 nm CW laser and a microscope objective with an NA of 0.82. The signal from the magnet was analyzed with a 750 mm long monochromator with a grating of 1800 grooves per mm and detected with a liquid-nitrogen-cooled CCD camera.

## Supporting Information Available

Crystal photographs, X-ray diffraction patterns, discussion of PL and reflectance spectra interpretation, analysis of polarization-resolved reflectance spectra, and reflectance spectra measured in the magnetic field (PDF)
