# Supplementary material for: Bright Excitonic Fine Structure in Metal-Halide Perovskites: From Two-Dimensional to Bulk
Source: J Am Chem Soc. 2024 Feb 7;146(7):4687–94. doi: 10.1021/jacs.3c11957 (PMC10885139; doi:10.1021/jacs.3c11957)
Supplement: Supplementary file 1 — ja3c11957_si_001.pdf [file ja3c11957_si_001.pdf]

Bright Excitonic Fine Structure in Metal Halide Perovskites: From 2D to Bulk - SI

Katarzyna Posmyk,[†,‡] Natalia Zawadzka,[¶] Łucja Kipczak,[¶] Mateusz Dyksik,[†] Alessandro Surrente,[†] Duncan K. Maude,[¶,‡] Tomasz Kazimierczuk,[¶] Adam Babiński,[¶] Maciej R. Molas,[¶] Wakul Bumrungsan,[§] Chanisara Chooseng,[‖] Watcharaphol Paritmongkol,[⊥,#,§] William A. Tisdale,[⊥] Michał Baranowski,[*,†] and Paulina Plochocka[*,†,‡]

†*Department of Experimental Physics, Faculty of Fundamental Problems of Technology, Wroclaw University of Science and Technology, Wroclaw 50-370, Poland*

‡*Laboratoire National des Champs Magnétiques Intenses, EMFL, CNRS UPR 3228, Université Grenoble Alpes, Université Toulouse, Université Toulouse 3, INSA-T, 38042 Grenoble and 31400 Toulouse, France*

¶*Institute of Experimental Physics, Faculty of Physics, University of Warsaw, 02-093 Warsaw, Poland*

§*Department of Materials Science and Engineering, School of Molecular Science and Engineering, Vidyasirimedhi Institute of Science and Technology (VISTEC), Rayong 21210, Thailand*

‖*Department of Chemical and Biomolecular Engineering, School of Energy Science and Engineering, Vidyasirimedhi Institute of Science and Technology (VISTEC), Rayong 21210, Thailand*

⊥*Department of Chemical Engineering, Massachusetts Institute of Technology, Cambridge, Massachusetts 02139, United States*

#*Department of Chemistry, Massachusetts Institute of Technology, Cambridge, Massachusetts 02139, United States*

E-mail: michal.baranowski@pwr.edu.pl; paulina.plochocka@lncmi.cnrs.fr

Crystal photographs

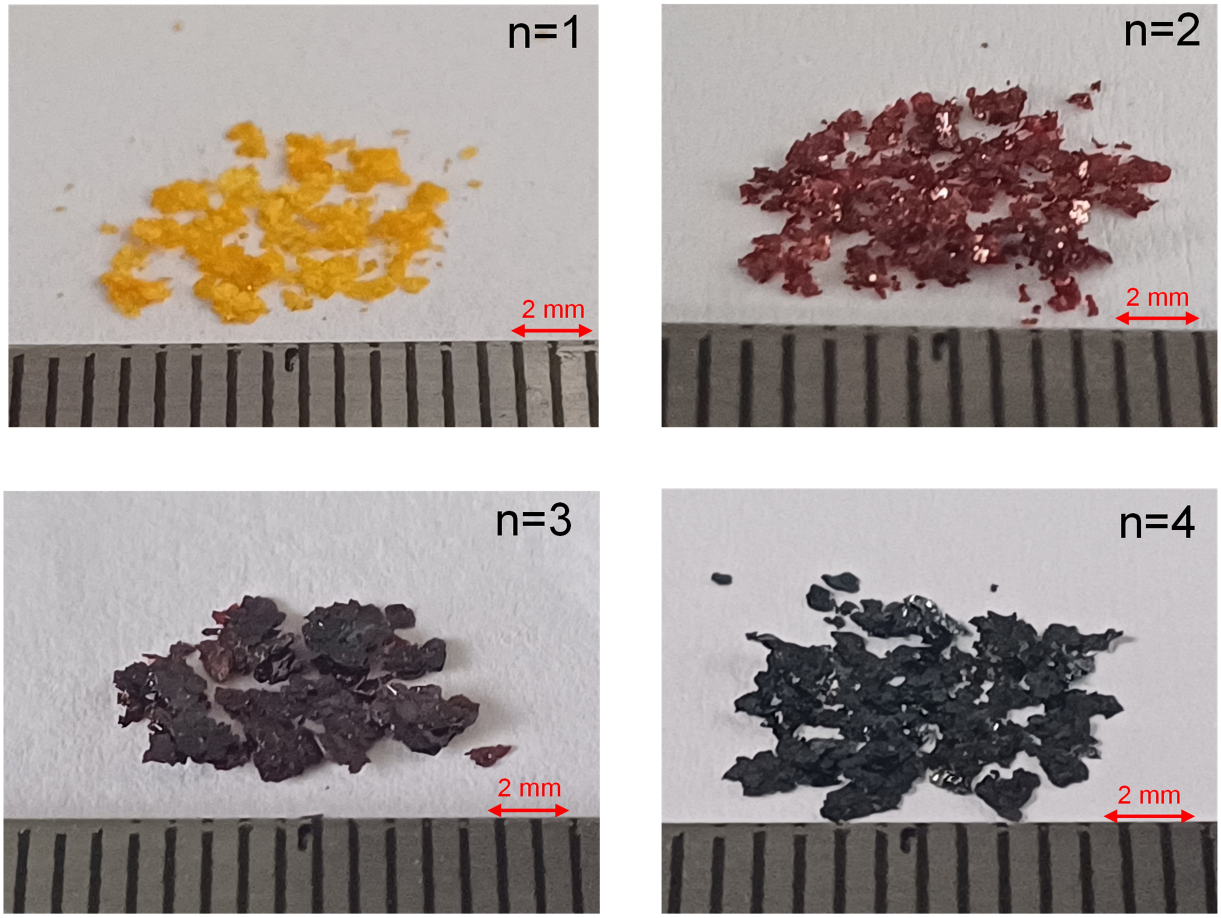

Figure S1: Photographs of $PEA_2(MA)_{n-1}Pb_nI_{3n+1}$ 2D perovskite with $n = 1, 2, 3,$ and 4 synthesized by cooling induced crystallization method.

X-ray diffraction patterns

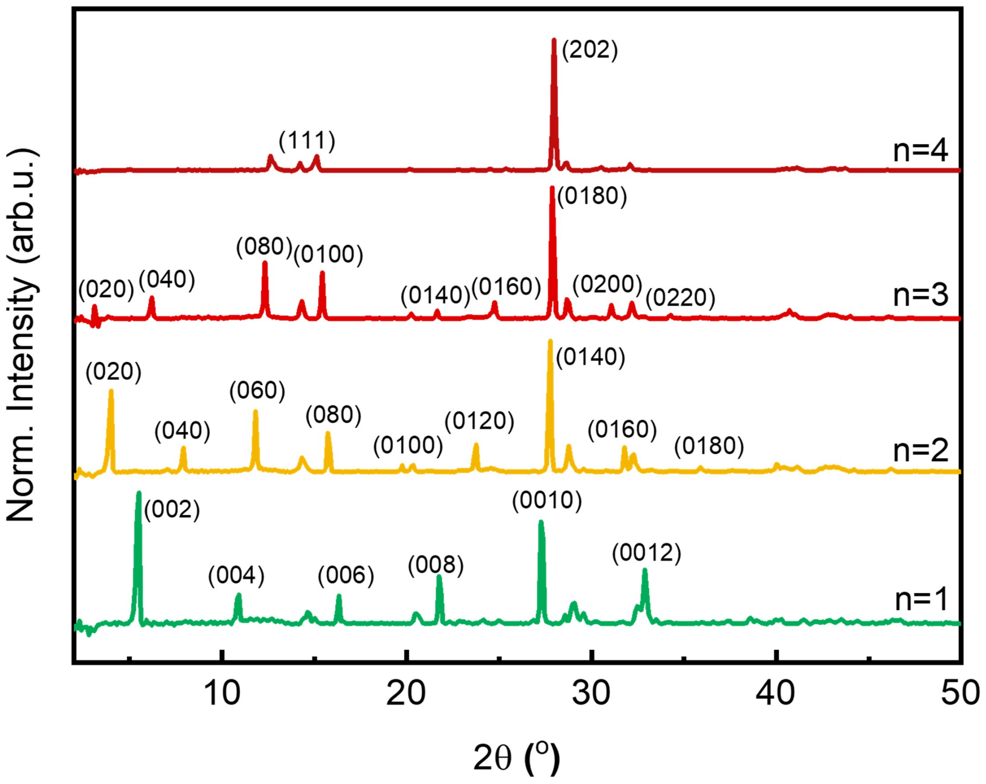

Figure S2: X-ray diffraction patterns of $PEA_2(MA)_{n-1}Pb_nI_{3n+1}$ 2D perovskite with $n = 1, 2, 3$, and 4

Disscusion of PL and R spectra interpretation

The particularly complex PL response of 2D perovskites (or generally of metal-halide perovskites) is a subject of ongoing debate in literature and many different assignments of complex PL spectrum can be found.[1–9] Therefore our analysis and conclusions presented in this paper are based on the analysis of the reflectance spectra.

It is crucial to emphasize that absorption and reflectivity spectra are predominantly sensitive to the high density of states (van Hove singularities), probing optical transitions between extended states, namely free excitonic transitions.[10] The consensus in the scientific community is that absorption-like techniques unequivocally probe the energy associated with

free excitonic transitions[11–14] and this is the most straightforward method for this purpose (as it is simultaneously not sensitive to localized states). At the same time, PL response remains inconclusive in this regard, primarily due to carrier thermalization and localization. This phenomenon results in the PL response probing the lowest energetic states, often trapping states which form a tail in the density of states.[10,15] In simpler terms, the PL response can be viewed as a convolution of the density of states and the thermal distribution of carriers/excitons. Consequently, the complexity of the PL response is attributed to this convolution, rendering the conclusive interpretation of free excitonic transitions challenging as often signal related to the trapping state is much stronger than free exciton recombination.[4,10,14–18] Moreover, the PL spectra can be affected by different excitonic complexes like biexciton for instance.[19]

Regarding our assignment of PL peak to free exciton transitions it is primarily based on the energy position of recombining species, aligning with the energy of excitonic transitions observed in reflectance spectra (equivalent to absorption spectra). The origin of extra redshifted PL peaks can have a threefold nature: (i) point defects,[3] (ii) local lattice reconfiguration induced by the cooling resulting from soft lattice and anharmonicity.[1,2,9] (iii) Large polaron formation/dressing of photoinduced carriers by the dynamic reconfiguration of lattice due to electronic excitation.[4,8?] However, at this moment we cannot provide any more precise assignment for the redshifted PL spectra part.

Polarisation resolved reflectance measurements

To extract the value of bright exciton fine structure splitting we use the fact that the line shape of our reflectance data is almost unchanged when measured with different polarization analyzer orientations as shown in Fig. S3-S5 (a). In Fig. S3-S5(b) we show ratio spectra (open symbols), *i.e.*, reflectance spectra measured at different analyzer orientations divided by the spectra taken at a selected analyzer orientation. Due to the shift of the transition

energy for different analyzer orientations, the ratio spectra (panel (b)) have a characteristic sharp feature with an amplitude, width and position which depend sensitively on the energy shift with respect to the selected reference spectrum. We fit the ratio spectra with:

$$\frac{R_\alpha(E)}{R_0(E)} = \frac{AR_0(E+\Delta E)+B}{R_0}, \tag{1}$$

where $R_0(E)$ and $R_\alpha(E)$ are the reference spectrum and the spectrum measured at a given orientation of the analyzer. A and C are constants, which take into account any possible change of the reflectance amplitude or background. ΔE is the shift of the reflectance resonance at a given angle with respect to reference spectra. To determine the value of ΔE, we fit the experimental curves using the least-squares method. As can be seen from panel (b), the agreement with the experiment is very good. Importantly, this method allows us to precisely determine the shift of exciton transition. The extracted shifts as a function of polarization angle are summarized in panel (c). In all cases, extracted shifts can be fitted with $\delta\cos^2(\alpha+\phi)$ function, which directly yields the value of the splitting of two in-plane bright states δ.

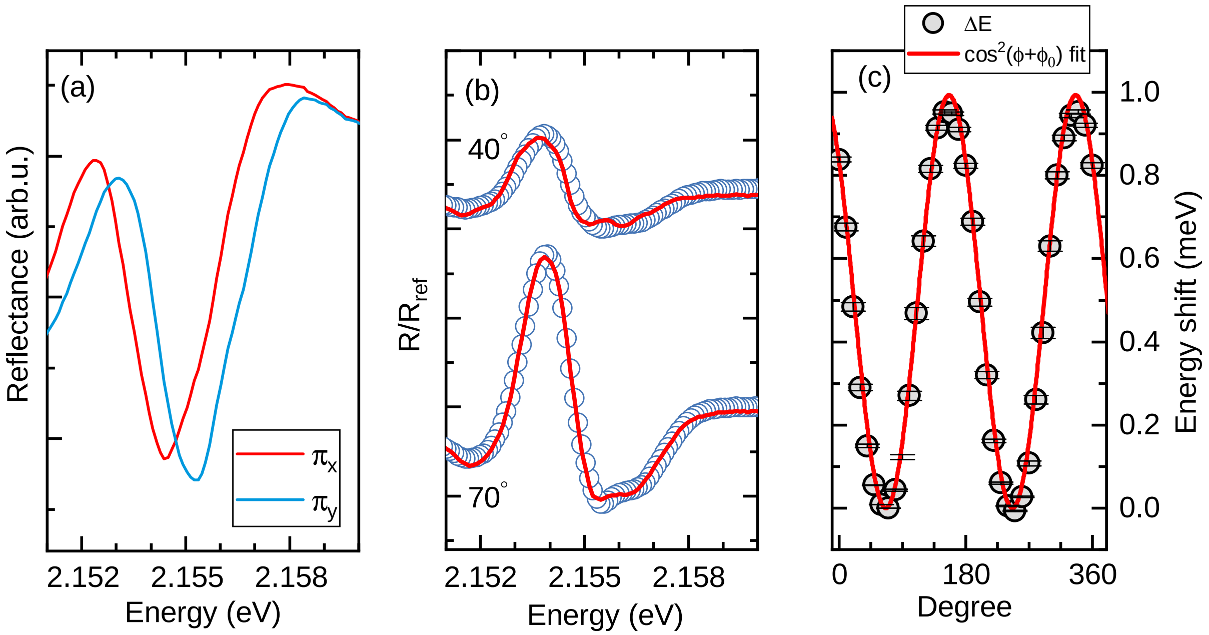

Figure S3: (a) Reflectance spectrum measured in two orthogonal linear polarizations from the sample with $n = 2$, showing clear splitting between two bright states of the exciton fine structure. (b) Fitting example and (c) extracted energy difference between two features in reflectance, from the differential method described in the main text and in detail in.[17] The black line is a $\cos^2(x)$ fit to the data points.

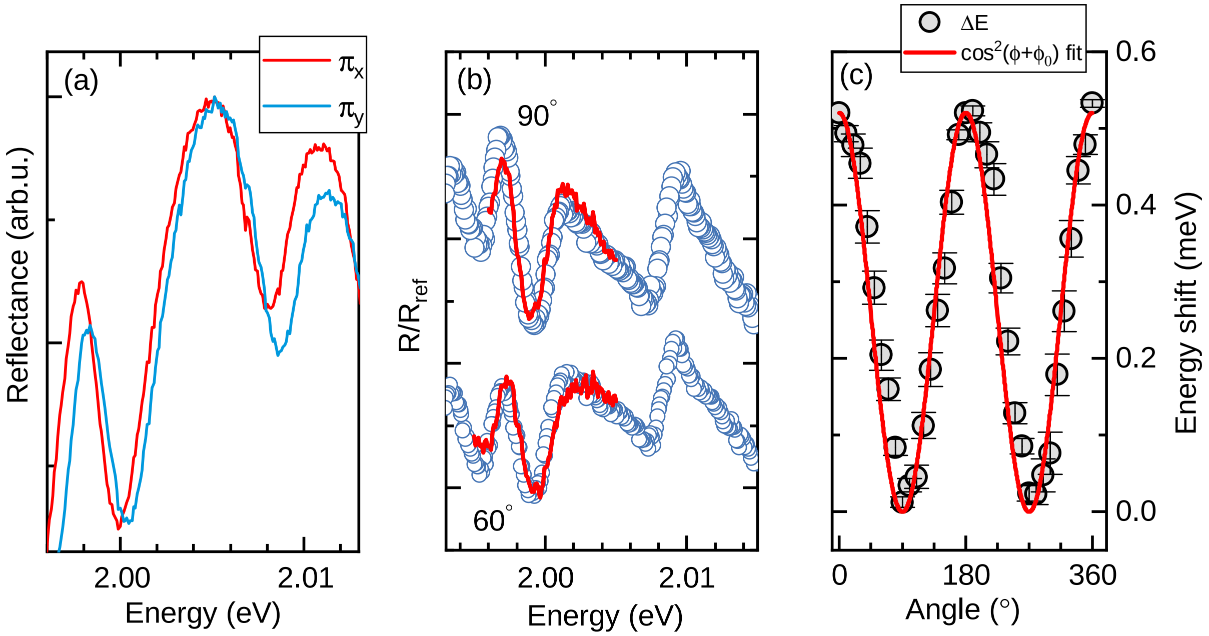

Figure S4: (a) Reflectance spectrum measured in two orthogonal linear polarizations from the sample with $n = 3$, showing clear splitting between two bright states of the exciton fine structure. (b) Fitting example and (c) extracted energy difference between two features in reflectance, from the differential method described in the main text and in detail in.[17] The black line is a $\cos^2(x)$ fit to the data points.

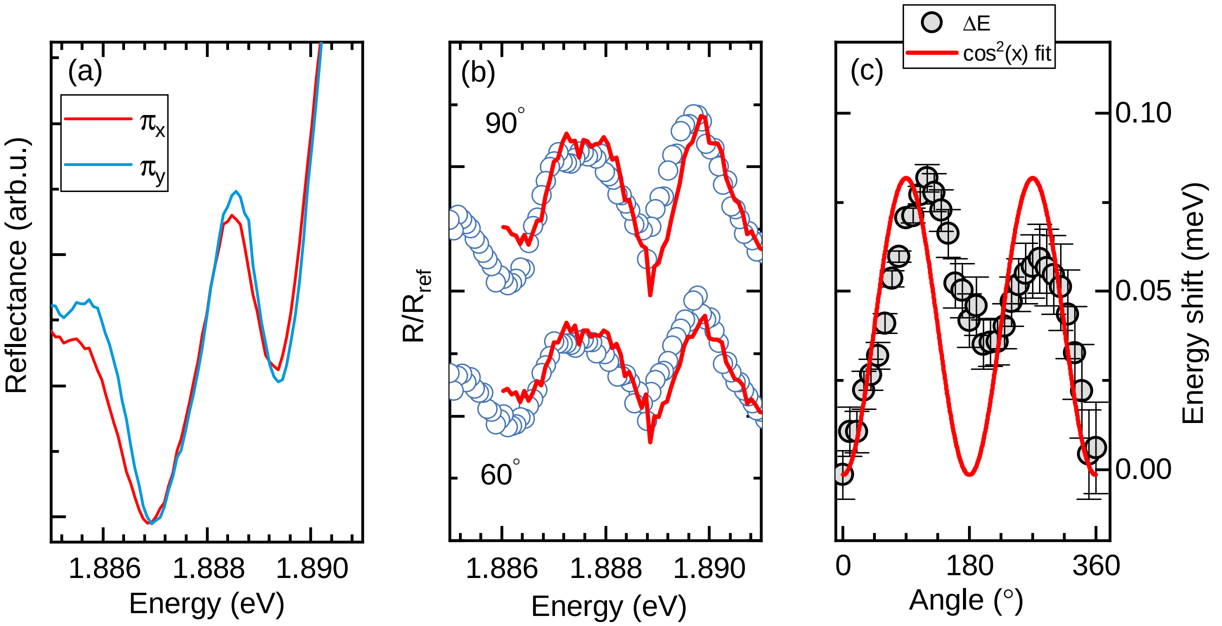

Figure S5: (a) Reflectance spectrum measured in two orthogonal linear polarizations from the sample with $n = 4$, showing clear splitting between two bright states of the exciton fine structure. (b) Fitting example and (c) extracted energy difference between two features in reflectance, from the differential method described in the main text and in detail in.[17] The black line is a $\cos^2(x)$ fit to the data points.

Magnetic field reflectance measurements

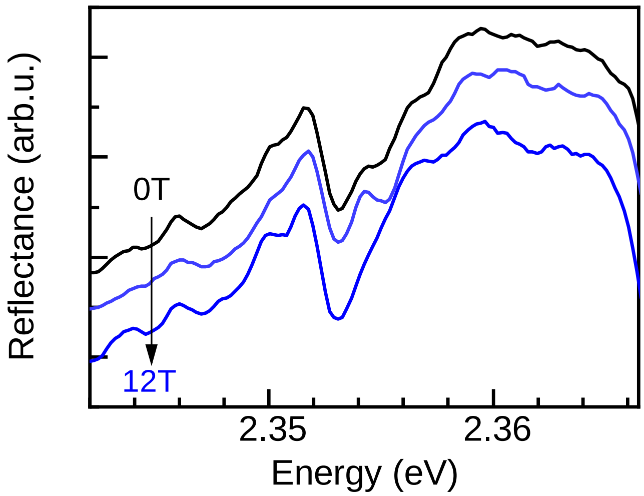

Figure S6: Reflectance spectra from the $n = 1$ sample for different magnetic fields measured in one circular polarisation. Features between 2.351 - 2.355 eV originate from two bright in plane excitonic transitions. As the excitonic states are mixing, their degree of circular polarisation increases. This results in the suppression of the signal originating from one of the states (here - energetically higher), whereas another one becomes more prominent (energetically lower).

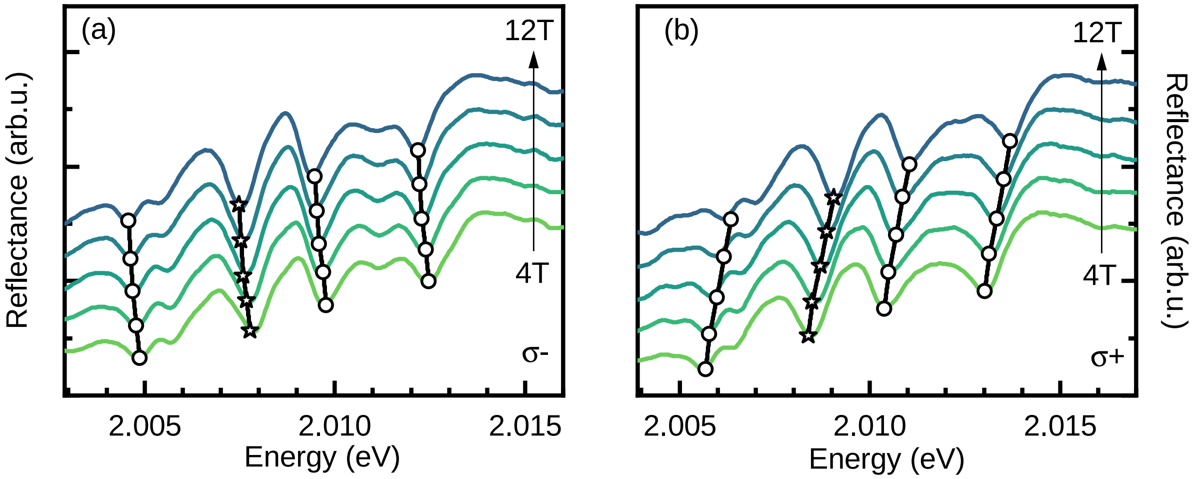

Figure S7: Reflectance spectra from the $n = 3$ sample for different magnetic fields together with the indicated shift of the excitonic transition. Panels (a) and (b) show the two different measured polarisations. The energy of the transition was taken from the feature indicated by stars.

To extract the values of the energy shift of the states in the magnetic field, we have adopted the following procedure. We measured the magnetoreflectance spectra in two orthogonal circular polarisations, from three different spots on the sample, in order to reduce the influence of the sample inhomogeneity on our results. We extracted the energy of the transition from the reflectance derivative spectrum minima. Then, we normalised the energy values by the energy at the zero fields, obtaining the absolute shift from zero-field values. Lastly, we took the mean values of energy shifts from three different spots, and fit them with Eq. (2).

$$E_{Y/X}(B) = \frac{1}{2}\left[(E_Y + E_X) \pm \sqrt{\delta^2 + g_B^2 \mu_B^2 B^2}\right] + c_0 B^2 \qquad (2)$$

We are aware that the reflectance spectrum is particularly complex for samples with $n = 3$ and $n = 4$. We notice that all of the features present the same spectral shift in the magnetic field, as shown in Figs. S7 and S8. This observation points to the phonon replicas origin of these features deriving from the same excitonic transition – zero phonon line.[20] Importantly, as long as all these lines shift in the same way as a function of the magnetic field or of the polarization detection angle, which is the case, their exact origin is not crucial

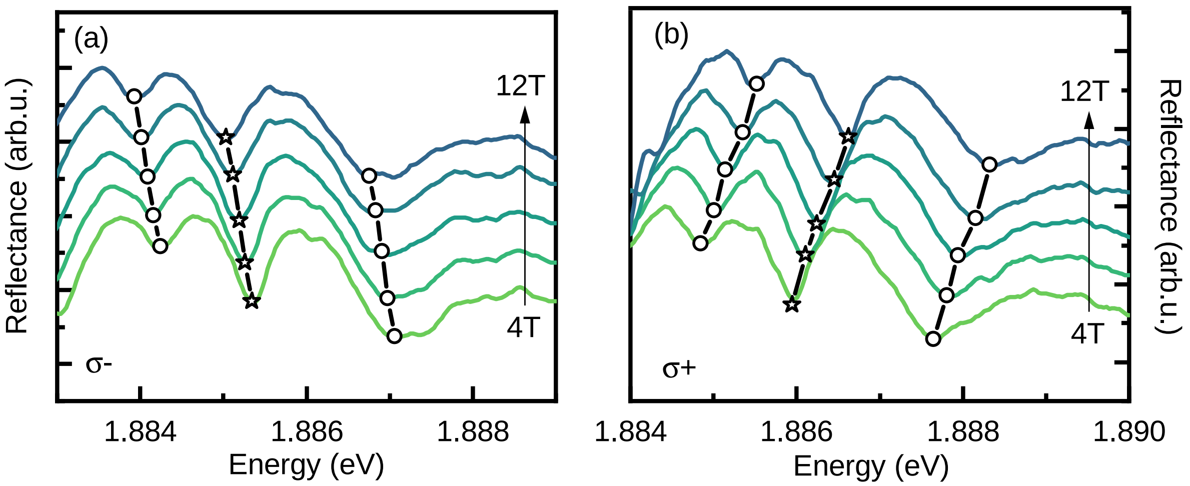

Figure S8: Reflectance spectra from the $n = 4$ sample for different magnetic fields together with indicated shift of the excitonic transition. Panels (a) and (b) show the two different measured polarisations. The energy of the transition was taken from the feature indicated by stars.

for our analysis. We indeed focus on the relative shifts, not on the absolute energy value of the excitonic transition. Moreover, if they represent different excitonic species, their shift should be different, which is not the case.

References

(1) Dar, M. I.; Jacopin, G.; Meloni, S.; Mattoni, A.; Arora, N.; Boziki, A.; Zakeeruddin, S. M.; Rothlisberger, U.; Grätzel, M. Origin of unusual bandgap shift and dual emission in organic-inorganic lead halide perovskites. *Science advances* **2016**, *2*, e1601156.

(2) Zuri, S.; Shapiro, A.; Kronik, L.; Lifshitz, E. Uncovering Multiple Intrinsic Chiral Phases in (PEA) 2PbI4 Halide Perovskites. *The Journal of Physical Chemistry Letters* **2023**, *14*, 4901–4907.

(3) Jin, H.; Debroye, E.; Keshavarz, M.; Scheblykin, I. G.; Roeffaers, M. B.; Hofkens, J.; Steele, J. A. It's a trap! On the nature of localised states and charge trapping in lead halide perovskites. *Materials Horizons* **2020**, *7*, 397–410.

(4) Hurtado Parra, S.; Straus, D. B.; Fichera, B. T.; Iotov, N.; Kagan, C. R.; Kikkawa, J. M.

Large exciton polaron formation in 2D hybrid perovskites via time-resolved photoluminescence. *ACS nano* **2022**, *16*, 21259–21265.

(5) Guo, Y.; Yaffe, O.; Hull, T. D.; Owen, J. S.; Reichman, D. R.; Brus, L. E. Dynamic emission Stokes shift and liquid-like dielectric solvation of band edge carriers in lead-halide perovskites. *Nature Communications* **2019**, *10*, 1175.

(6) Kahmann, S.; Duim, H.; Fang, H.-H.; Dyksik, M.; Adjokatse, S.; Rivera Medina, M.; Pitaro, M.; Plochocka, P.; Loi, M. A. Photophysics of two-dimensional perovskites—learning from metal halide substitution. *Advanced Functional Materials* **2021**, *31*, 2103778.

(7) Canet-Albiach, R.; Krecmarova, M.; Bailach, J. B.; Gualdrón-Reyes, A. F.; Rodríguez-Romero, J.; Gorji, S.; Pashaei-Adl, H.; Mora-Seró, I.; Martinez Pastor, J. P.; Sánchez-Royo, J. F.; others Revealing Giant Exciton Fine-Structure Splitting in Two-Dimensional Perovskites Using van der Waals Passivation. *Nano Letters* **2022**, *22*, 7621–7627.

(8) Tao, W.; Zhang, C.; Zhou, Q.; Zhao, Y.; Zhu, H. Momentarily trapped exciton polaron in two-dimensional lead halide perovskites. *Nature Communications* **2021**, *12*, 1400.

(9) Shao, Y.; Gao, W.; Yan, H.; Li, R.; Abdelwahab, I.; Chi, X.; Rogée, L.; Zhuang, L.; Fu, W.; Lau, S. P.; others Unlocking surface octahedral tilt in two-dimensional Ruddlesden-Popper perovskites. *Nature Communications* **2022**, *13*, 138.

(10) Bastard, G. Wave mechanics applied to semiconductor heterostructures. **1990**,

(11) Weisbuch, C.; Dingle, R.; Gossard, A.; Wiegmann, W. Optical characterization of interface disorder in GaAs-Ga1-xAlxAs multi-quantum well structures. *Solid State Communications* **1981**, *38*, 709–712.

(12) Skolnick, M.; Tapster, P.; Bass, S.; Pitt, A.; Apsley, N.; Aldred, S. Investigation of InGaAs-InP quantum wells by optical spectroscopy. *Semiconductor science and technology* **1986**, *1*, 29.

(13) Kudrawiec, R.; Latkowska, M.; Baranowski, M.; Misiewicz, J.; Li, L.; Harmand, J. Photoreflectance, photoluminescence, and microphotoluminescence study of optical transitions between delocalized and localized states in GaN 0.02 As 0.98, Ga 0.95 In 0.05 N 0.02 As 0.98, and GaN 0.02 As 0.90 Sb 0.08 layers. *Physical Review B* **2013**, *88*, 125201.

(14) Kudrawiec, R.; Sęk, G.; Misiewicz, J.; Ishikawa, F.; Trampert, A.; Ploog, K. H. Localized and delocalized states in GaNAs studied by microphotoluminescence and photoreflectance. *Applied Physics Letters* **2009**, *94*.

(15) Pelant, I.; Valenta, J. *Luminescence spectroscopy of semiconductors*; OUP Oxford, 2012.

(16) Wright, A. D.; Milot, R. L.; Eperon, G. E.; Snaith, H. J.; Johnston, M. B.; Herz, L. M. Band-tail recombination in hybrid lead iodide perovskite. *Advanced Functional Materials* **2017**, *27*, 1700860.

(17) Baranowski, M.; Galkowski, K.; Surrente, A.; Urban, J.; Kłopotowski, Ł.; Mackowski, S.; Maude, D. K.; Ben Aich, R.; Boujdaria, K.; Chamarro, M.; others Giant fine structure splitting of the bright exciton in a bulk MAPbBr3 single crystal. *Nano Letters* **2019**, *19*, 7054–7061.

(18) Baranowski, M.; Urban, J. M.; Zhang, N.; Surrente, A.; Maude, D.; Andaji-Garmaroudi, Z.; Stranks, S.; Plochocka, P. Static and dynamic disorder in triple-cation hybrid perovskites. *The Journal of Physical Chemistry C* **2018**, *122*, 17473–17480.

(19) Fang, H.-H.; Yang, J.; Adjokatse, S.; Tekelenburg, E.; Kamminga, M. E.; Duim, H.; Ye, J.; Blake, G. R.; Even, J.; Loi, M. A. Band-edge exciton fine structure and exci-

ton recombination dynamics in single crystals of layered hybrid perovskites. *Advanced Functional Materials* **2020**, *30*, 1907979.

(20) Urban, J. M.; Chehade, G.; Dyksik, M.; Menahem, M.; Surrente, A.; Trippé-Allard, G.; Maude, D. K.; Garrot, D.; Yaffe, O.; Deleporte, E.; others Revealing Excitonic Phonon Coupling in (PEA) 2 (MA) n- 1Pb n I3 n+ 1 2D Layered Perovskites. *The Journal of Physical Chemistry Letters* **2020**, *11*, 5830–5835.
